# LodgeNet: an automated framework for precise detection and classification of wheat lodging severity levels in precision farming

**DOI:** 10.3389/fpls.2023.1255961

**Published:** 2023-11-28

**Authors:** Nisar Ali, Ahmed Mohammed, Abdul Bais, Jatinder S. Sangha, Yuefeng Ruan, Richard D. Cuthbert

**Affiliations:** ^1^ Faculty of Engineering and Applied Science, University of Regina, Regina, SK, Canada; ^2^ Swift Current Research and Development Centre, Agriculture and Agri-Food Canada, Swift Current, SK, Canada

**Keywords:** wheat lodging, classification, multi-spectral imaging, Unmanned Aerial Vehicle, deep learning

## Abstract

Wheat lodging is a serious problem affecting grain yield, plant health, and grain quality. Addressing the lodging issue in wheat is a desirable task in breeding programs. Precise detection of lodging levels during wheat screening can aid in selecting lines with resistance to lodging. Traditional approaches to phenotype lodging rely on manual data collection from field plots, which are slow and laborious, and can introduce errors and bias. This paper presents a framework called ‘LodgeNet,’ that facilitates wheat lodging detection. Using Unmanned Aerial Vehicles (UAVs) and Deep Learning (DL), LodgeNet improves traditional methods of detecting lodging with more precision and efficiency. Using a dataset of 2000 multi-spectral images of wheat plots, we have developed a novel image registration technique that aligns the different bands of multi-spectral images. This approach allows the creation of comprehensive RGB images, enhancing the detection and classification of wheat lodging. We have employed advanced image enhancement techniques to improve image quality, highlighting the important features of wheat lodging detection. We combined three color enhancement transformations into two presets for image refinement. The first preset, ‘Haze & Gamma Adjustment,’ minimize atmospheric haze and adjusts the gamma, while the second, ‘Stretching Contrast Limits,’ extends the contrast of the RGB image by calculating and applying the upper and lower limits of each band. LodgeNet, which relies on the state-of-the-art YOLOv8 deep learning algorithm, could detect and classify wheat lodging severity levels ranging from no lodging (Class 1) to severe lodging (Class 9). The results show the mean Average Precision (mAP) of 0.952% @0.5 and 0.641% @0.50-0.95 in classifying wheat lodging severity levels. LodgeNet promises an efficient and automated high-throughput solution for real-time crop monitoring of wheat lodging severity levels in the field.

## 1 Introduction

Wheat is a crucial food crop that provides essential nutrients to consumers worldwide. To ensure that sufficient food is available to the growing world’s population, it is essential to maintain wheat yield and quality by managing the limiting factors that affect its growth and production, such as lodging (Jiang et al., 2022; Yu et al., 2022). Lodging in wheat may cause a yield reduction from > 50% depending on the extent of damage, affecting kernel numbers per spike, inducing sprout damage in grain, interfering with harvesting, and deteriorating post-harvest grain quality, thereby lowering the wheat market grade (Fischer and Stapper, 1987; Berry and Spink, 2012; Pin˜era-Chavez et al., 2016). Wheat lines with lodging resistance are more desirable, and a target trait in breeding (Shah et al., 2019). Various stakeholders, including breeders, agronomists, plant physiologists, farmers, and crop insurance personnel, require accurate and timely assessment of wheat lodging to mitigate its impact on wheat growth, production and marketability (Kang et al., 2023; Sun et al., 2023). Lodging resistance would help to minimize yield losses and maintain crop quality (Yang et al., 2021). Developing efficient and reliable methods for lodging detection is, therefore, crucial.

Traditional methods for detecting wheat lodging are manual that rely on visual and subjective rating of the plants’ stature, which is highly laborious phenotyping and a time consuming activity (Ali et al., 2023; Modi et al., 2023; Zaji et al., 2023). These methods are more prone to error depending on a number of factors, including the expertise of the person rating the lodging and, the time of rating after the lodging, to some extent, affected by extreme environmental conditions during the assessment, affecting the accuracy and reliability of the results (Ali et al., 2019a). Emerging technologies such as remote sensing, Unmanned Aerial Vehicles (UAVs), and Artificial Intelligence (AI) hold great promise for non-invasive, rapid, and accurate monitoring of crop lodging (Zhang et al., 2020). Integrating these technologies with traditional methods could overcome the limitations of the current manual approach, enabling more efficient, objective, and reliable detection of wheat lodging (Wen et al., 2022). Among various technologies, UAVs have great potential in improving the efficiency and reliability of wheat lodging detection, providing the opportunity to improve high throughout phenotyping.

Recent years have witnessed significant progress in crop monitoring, using more advanced UAV platforms and information processing technologies (Chauhan et al., 2019a). UAVs are effective, low-cost, and flexible tools in precision agriculture, offering great potential in plant breeding, especially for screening thousands of lines in a shorter time. The use of Machine Learning (ML) techniques together with Red, Green, and Blue (RGB) images has significantly improved crop breeding phenomics by enabling the selection of various traits like crop lodging information and area (Liu et al., 2018; Asad and Bais, 2020a). For example, (Fang et al., 2021) collected RGB images using a UAV platform post-wheat lodging and combined Digital Surface Model (DSM) images and Excess Green Vegetation Index (EXG) to generate “DSM + RGB” and “DSM + EXG” fusion images. The researchers then applied maximum likelihood and random forest methods to the images, achieving an impressive 93.75% accuracy in extracting information on wheat lodging areas from the “DSM + RGB” fusion images. Similarly, (Li et al., 2019) used a comprehensive feature model based on two individual features of wheat lodging extracted from UAV RGB images and a K-means algorithm to develop a multi-temporal lodging area extraction method. In addition, (Chauhan et al., 2019b) utilized UAV multi-spectral images to differentiate among various categories of lodged wheat plants, achieving an overall accuracy rate of 90% through a multi-resolution segmentation algorithm and the nearest neighbor classification algorithm. (Cao et al., 2021) proposed a hybrid algorithm founded on a watershed algorithm and adaptive threshold segmentation to extract the lodging in wheat. Despite the high recognition accuracy of ML techniques in UAV-based monitoring for lodging, challenges in practical application continue to exist, primarily due to the need to select precise features of the trait to ensure the effectiveness of these techniques (Das and Bais, 2021; Yu et al., 2023). This underscores the importance of alternative methods, such as advanced remote sensing technologies, which could enhance the capabilities of UAV-based monitoring in the lodging detection process.

Satellite-based remote sensing technologies have been widely adopted for detecting wheat lodging (Shu et al., 2023). However, their effectiveness is limited due to low spatiotemporal resolution and weak spectral differences between lodged and non-lodged crops (Asad and Bais, 2020b). Aerial remote sensing can provide higher spatial and temporal resolution, but it is more complex and expensive. In contrast, UAVs are cost-effective, flexible, and high-resolution alternatives to satellite and aerial systems for detecting wheat lodging (Chauhan et al., 2021). Despite their potential, there is a need for methodological advancements in capturing and analyzing data, particularly in generating and utilizing DSMs for enhanced information. One approach to improve the quality of the information obtained from DSMs is to use fusion images based on RGB and DSM, but there is no consensus on which information is best to fuse. DSMs alone may not capture subtle details related to lodging, especially when the lodging severity is low. On the other hand, RGB images capture the visual appearance of the crop canopy, which includes colour and texture variations. By fusing RGB images with DSMs, it leverages the complementary information offered by these two sources (Al-Najjar et al., 2019). Moreover, extracting lodging information still presents challenges that need to be addressed to increase the accuracy and reliability of wheat lodging detection (Xie et al., 2021).

Further evaluation is required to determine the optimal mission height for UAV-based wheat lodging detection (Dai et al., 2023). Diverse image types, such as RGB, multi-band spectral and thermal images, have been utilized to develop classification algorithms in various crops (Vargas et al., 2019; Chauhan et al., 2020a). However, there is a need for additional research to identify specific challenges, such as the impact of varying image types and classification algorithms on the precision of wheat lodging detection in UAV-based studies.

Manual cropping of images for dataset creation can be time-consuming, prone to human error, and may lead to inconsistent results (Ali et al., 2019b; Yu et al., 2023). Thus, a semi-automatic or fully automatic approach is necessary to streamline and standardize dataset creation. The fusion of emerging ML and DL algorithms with remote sensing technologies has shown the potential to enhance the efficiency and accuracy of wheat lodging detection (Jiang et al., 2022). However, further studies are needed to measure the impact of automation on dataset creation time, the accuracy of lodging detection, and the consistency of results. Feature extraction and classification can also aid in lodging detection (Ullah et al., 2021).

Detecting crop lodging involves a classification task that requires gathering features from image datasets and applying ML algorithms (Zhang et al., 2022). DL models can automate the feature extraction and classification process, but their effectiveness for wheat lodging detection is still poorly explored. Few studies (e.g. (Chauhan et al., 2020b)) have directly compared the performance of DL and traditional ML algorithms in detecting wheat lodging, indicating a gap in the literature and a need for more comprehensive studies. DL models, specifically Convolutional Neural Networks (CNNs), can extract detailed image features but often require substantial computational resources and exhibit complexity, hindering their large-scale, real-time use (Masuda et al., 2013; Wang et al., 2019; Raja et al., 2020; Setyawan et al., 2020; He et al., 2021). Therefore, it is worth exploring the specific role of DL, particularly its strengths and potential limitations, in tackling lodging recognition challenges.

The paper aims to tackle the above-mentioned issues by addressing the following objectives: (1) collect a vast dataset of multi-spectral images of wheat plots, (2) focus on developing an image registration technique for multi-spectral images, (3) enhance the image quality to improve the detection of wheat lodging, and (4) build an automated framework named ‘LodgeNet’ that can detect and classify the severity of wheat lodging into nine different classes using advanced deep learning techniques. This research work is the first to classify wheat lodging severity levels into different classes, ranging from no lodging (Class 1) to severe lodging (Class 9).

## 2 Materials and methods

To develop the models for lodging detection, the data was collected in a field set for evaluating different wheat lines. The data sets were pre-processed, before applying a DL model to identify severity levels of wheat lodging, to assess its practical application in wheat fields.

### 2.1 Experimental field and data collection

**Figure 1:**
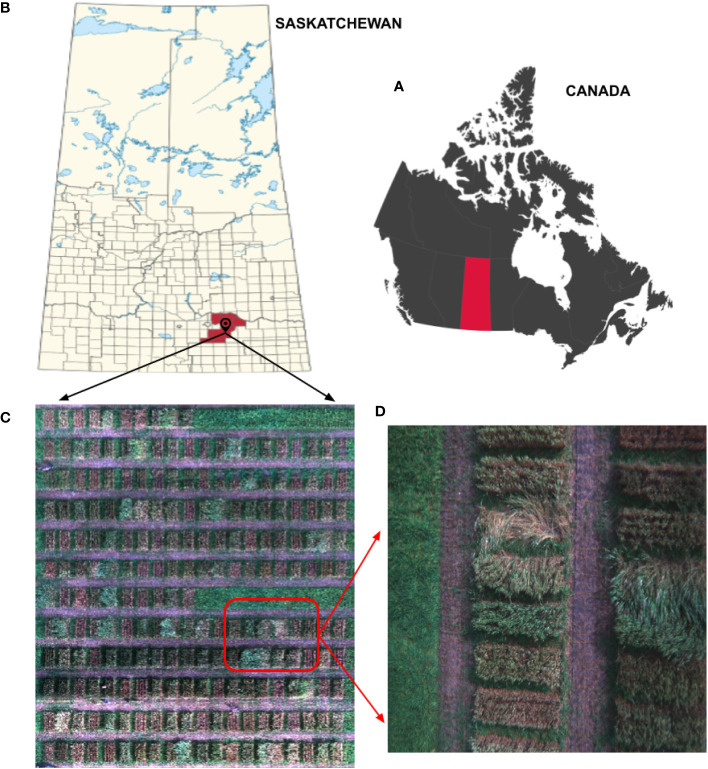
Location of UAV imaging area, study site, and lodging sample. **(A)** Canada map locating Saskatchewan province. **(B)** Indian Head Agricultural Research Foundation (IHARF). **(C)** Mosaic of the lodged field (experimental field). **(D)** An image of wheat lodging.

A panel of durum wheat [Triticum turgidum L. ssp. durum (Desf.) Husn.], comprising 110 diverse lines from Canadian and international collections, was used as a platform for this study. These lines differ in yield, height, lodging, and other grain quality parameters. Replicated field plots were seeded in 2022 using a randomized complete block design with two replications at Agriculture and Agri-Food Canada’s research farm in Indian Head, SK. The field plots at Indian Head (**Figure 1**) were seeded under dry-land conditions without artificial irrigation. Each plot measured 3*m* in length, and 1.2*m* in width was seeded with 1,200 seeds in four rows with a gap of 0.23*m* between rows. The gap between plots was filled by seeding two rows of winter wheat. Standard field practices were followed for fertilization, weed, disease, and pest control to minimize other factors in yield limitations. Wheat lines were evaluated for lodging, grain yield, quality, and other agronomic characteristics. Grain yield was calculated per plot and converted to a per-hectare basis using a conversion factor.

Lodging was rated using a 1-9 scale, where 1 is assigned to plants with the upright position in the plot with no sign of lodging, whereas 9 is used when more than 75% of plants in the plot are laid horizontally flat on the ground. **Table 1** presents an illustration of the evaluation process.

**Table 1:** Class-wise manual evaluation of wheat lodging.

Entry Tag #	Plot #	Yield (g/plot)	Lodging Class
23	2	1572.7	1
26	1	1126.4	2
90	13	1060.7	3
14	16	719.8	4
92	10	1331.2	5
88	58	937.6	6
89	82	963.9	7
82	96	446.9	8

**Figure 2:**
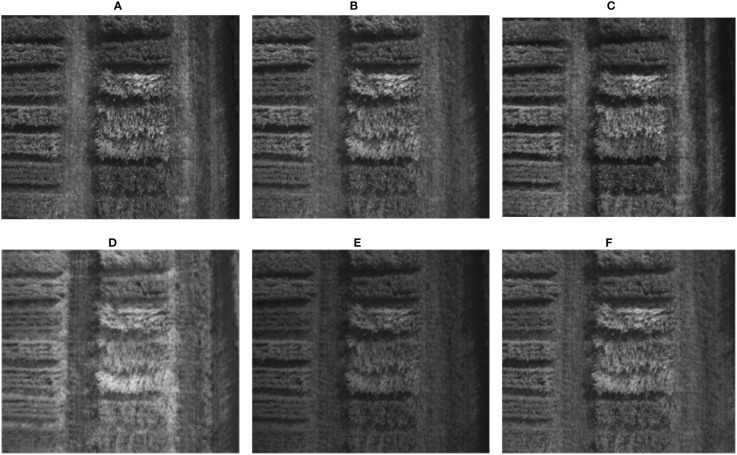
Six bands of the same image captured by the camera MicaSense RedEdge-P, **(A)** Blue (475 nm center, 32 nm bandwidth), **(B)** Green (560 nm center, 27nm bandwidth), **(C)** Red (668 nm center, 14 nm bandwidth), **(D)** Red Edge (717 nm center, 12nm bandwidth), **(E)** Near-IR (842 nm center, 57 nm bandwidth), **(F)** Panchromatic 634.5 nm center, 463 nm bandwidth.

In this study, we used the MicaSense RedEdge-P Camera for all experimental image data collection. MicaSense RedEdge-P Camera is a six-band multi-spectral sensor for agricultural and environmental monitoring applications. It captures images of the same region in six different spectrum bands: blue, green, red, red edge, near-infrared, and panchromatic (**Figure 2**). The study collected around 2000 aerial images taken from an altitude of 42 feet over the wheat plots in the field using UAV before the maturity stage. The panchromatic band of the RedEdge-P Camera, with a resolution of 2464 × 2056, covers the entire visible spectrum and provides a higher spatial resolution than the other bands, which have a resolution of 1456 × 1088. This allows for more detailed and precise imaging of the captured region. Six files are generated for each shot the camera takes, each containing the information captured by one of the six bands. The images are saved in a 16-bit TIFF format, ensuring high precision in the captured data. The Agisoft Metashape software (Agisoft Metashape, 2010; Kingsland, 2020) was used to obtain an orthomosaic of the wheat field, which was further processed using the Geographic Information System Application QGIS (QGIS, 2002; Moyroud and Portet, 2018).

### 2.2 Data pre-processing

Multi-spectral images captured by UAVs or other independent devices typically consist of multiple bands, each captured by a sensor and covering the same area. These bands are processed to create indexes such as the Normalized Difference Vegetation Index (NDVI) (Pettorelli, 2013), Visible Atmospherically Resistant Index (VARI) (Sewiko and Sagala, 2022), Green Atmospherically Resistant Index (GARI) (Sun, 2023), Surface Algal Bloom Index (SABI) (Alawadi, 2010), or combined into multi-band compositions. As physically separate sensors capture the bands, they must be aligned before generating multi-band compositions. The offset between them varies based on the physical distance between the sensors and the distance between the camera and the photographed objects (Abbas et al., 2020). The formulas for the four vegetation indices can be found in **Table 2**.

**Table 2:** Vegetation indices and their formulas.

Index	Formula
Normalized Difference Vegetation Index (NDVI)	$NDVI=\frac{NIR-R}{NIR+R}$
Visible Atmospherically Resistant Index	$\left(VARI=\frac{G-R}{G+R-B}\right)$
Green Atmospherically Resistant Index (GARI)	$GARI=\frac{NIR-(G-(B-R))}{NIR-(G+(B-R))}$
Surface Algal Bloom Index (SABI)	$SABI=\frac{NIR-R}{B+G}$

#### 2.2.1 Image registration

In this study, a technique for aligning the bands of multi-spectral images captured by separate sensors has been developed. This technique is based on image intensity and involves an iterative process that requires the specification of a pair of images, a metric, an optimizer, and a transformation type. The image similarity metric evaluates the accuracy of the registration process and produces a scalar value that indicates the degree of similarity between the two images. The optimizer determines the method for minimizing or maximizing the similarity metric, while the transformation type, describing the mathematical model that explains the geometric relationship between the fixed and moving images, specifies the 2-D transformation needed to align the misaligned image with the reference image. The process starts with the transformation type and an internally determined transformation matrix, which determines the specific image transformation applied to the moving image through bi-linear interpolation. The metric then compares the transformed moving image to the fixed image and calculates a metric value. Finally, the optimizer checks for a stop condition that could indicate the termination of the process. The process usually ends when the returns diminish, or the maximum number of iterations is reached. If there is no stop condition, the optimizer adjusts the transformation matrix and starts the next iteration. The algorithm for this method is detailed in **Algorithm 1**.

Algorithm 1 **Multi-spectral image registration and processing**

**Input:** Multi-Spectral Images (6-Bands)
**Output:** Alligned Bands with RGB Image
1: Specify ← (pair of images, metric, optimizer, transformation type)
2: **for** each iteration **do**
3:  Determine transformation matrix using transformation type
4:  Apply transformation to moving image using bi-linear interpolation
5:  Calculate metric value by comparing transformed moving image to fixed image
6:  **if** stop condition is met **then**
7:   Terminate process
8:  **else**
9:   Adjust transformation matrix
10:  **end if**
11: **end for**

The optimization techniques and parameters are established through experiments with images acquired from various locations during the flights. The alignment process involves pairing the images based on a reference band. Band 2 (Green, with a center wavelength of 560 nm and a bandwidth of 27 nm) is used as the reference band to align other bands, which results in optimal calibration. The transformation type ‘rigid’ produces the best results in the case of this study, and the optimizer and metric are configured based on the following parameters. Mathematically, the similarity metric is defined in (1), and the optimizer is outlined in (2).

• GrowthFactor = 1.002
• MaximumIterations = 500
• InitialRadius = 0.0002
• Epsilon = 0.0000015

(1) $$M(I_{ref},I_{mov},T)=\sum_{x,y}w(x,y),s(I_{ref}(x,y),I_{mov}(T(x,y)))$$

where:

- *I_ref_* and *I_mov_* are the fixed and moving images, respectively, *T* is the 2-D transformation
- *M*(*I_ref_, I_mov_, T*) represents the similarity metric, which is a scalar value that indicates the degree of similarity between the reference and moving images. It is calculated as the sum of the product of the weighting function w(x,y) and the similarity function *s*(*I_ref_* (*x,y*)*, I _mov_* (*T*(*x,y*))) evaluated at each pixel location (*x,y*) in the images.
- The similarity function *s*(*I_ref_* (*x,y*)*, I _mov_* (*T*(*x,y*))), defined in (3), measures the similarity between the intensity values of the reference and transformed moving images at each pixel location. This research employed a similarity function called Mutual Information (MI). The choice of similarity function depends on the specific application and the registered images’ characteristics.
- The weighting function w(x,y), described in (4), assigns higher or lower importance to certain regions of the images based on their characteristics or prior knowledge about the scene. This allows the registration process to focus on the images’ most informative regions and improves the alignment’s accuracy.

(2) $$T^{*}=\arg\;\underset{T}{\max}M\left(I_{ref},I_{mov},T\right)$$

(3) $$s(I_{ref}(x,y),I_{mov}(T(x,y)))=\sum_{i,j}p_{i,j}\log\frac{p_{i,j}}{p_{i}p_{j}}$$

where *p_i,j_* is the joint histogram of *I_ref_ andI_mov_* (*T*), and *p_i_* and *p_j_* are the marginal histograms of *I_ref_* and *I_mov_* (*T*), respectively.


(4) $$w(x,y)=e^{-\frac{{\left(x-x_{c}\right)}^{2}+{\left(y-y_{c}\right)}^{2}}{2\sigma^{2}}}$$

where (*x_c_,y_c_*) is the center of the image and *σ* controls the spread of the Gaussian.

**Figure 3:**
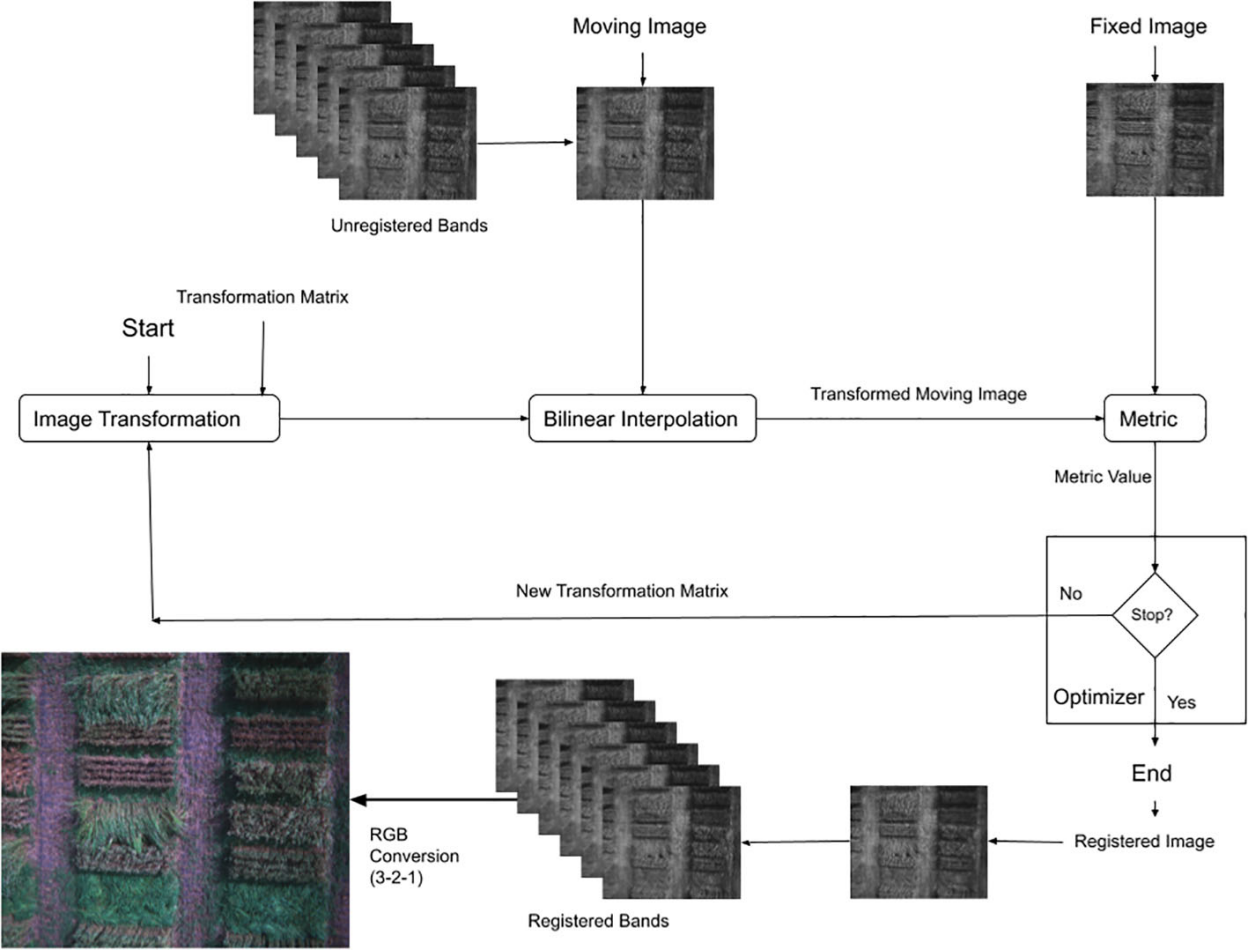
Process of multi-spectral images registration and processing to RGB images (3-2-1).

After performing the alignment of multi-spectral images using a reference band, a single RGB image was generated for detecting and categorizing wheat lodging severity. Generating the RGB images involves combining the aligned bands of the multi-spectral images into a single image with three channels: blue, green, and red. The outcome is a true color RGB image, commonly called a 3-2-1 image, due to the bands assigned to the red, green, and blue channels. These images are also known as RGB3-2-1 images. **Figure 3** provides a visual representation of this process.

#### 2.2.2 Image enhancement and composition

To make the spectral features in multi-spectral images more visible, additional images with enhanced colors were created for each composition. Three different color enhancement transformations were utilized and combined into two presets. The first preset, Haze & Gamma Adjustment, involved reducing atmospheric haze and adjusting the Gamma. The formula for adjusting Haze & Gamma is presented in (5).

The second preset, Stretching Contrast Limits, helped to calculate each band’s upper and lower limits and used them to stretch the image’s contrast. The formula for stretching contrast limits is given by (6).


(5) $$\begin{array}{c} a_{c}=0.25\cdot percentile\;(I_{in}(:,:,c),1)\\ t_{c}=(I_{in}(x,y,c)>a_{c})\cdot (1+\alpha_{c}\cdot (I_{in}(x,y,c)-a_{c})){)}^{-\beta_{c}}\\ b_{c}=\frac{1}{n}\sum_{x,y}(I_{in}(x,y,c)-a_{c})\cdot t_{c}^{\gamma_{c}}\\ I_{out}(x,y,c)=b_{c}\cdot t_{c}^{\gamma_{c}} \end{array}$$

where:

- *I_in_* is the input image.
- *I_out_* is the output image after Haze & Gamma Adjustment.
- *c* denotes the color channel (e.g., *c* = 1 for red, *c* = 2 for green, *c* = 3 for blue).
- *α_c_*, *β_c_*, *γ_c_* are the parameters controlling the intensity of haze reduction, the shape of the haze adjustment curve, and the gamma correction, respectively.
- *a_c_* is the dark channel value for color channel *c*, computed as the 1% percentile of *I_in_* in that channel.
- *t_c_* is the haze adjustment term for color channel *c*.
- *b_c_* is the brightness adjustment term for color channel *c*, computed as the average of *I_in_* after haze adjustment.
- percentile computes the nth percentile of a set of values.
- The values for the Haze & Gamma Adjustment preset are *α_c_ *= 0.2, *β_c_* = 0.7 for haze reduction, and *γ_c_* = 0.6 for gamma correction.

(6) $$I_{out}\left(x,y,c\right)=\frac{I_{in}\left(x,y,c\right)-I_{min,c}}{I_{max\text{, }c}-I_{min\text{, }c}}\cdot 255$$

where:

- *I_in_* is the input image.
- *I_out _* is the output image after Stretching Contrast Limits.
- *c* denotes the color channel (e.g., *c* = 1 for red, *c* = 2 for green, *c* = 3 for blue).
- *I_min,c _* and *I_max,c_* are the lower and upper limits of color channel *c*, respectively.

Each image band’s minimum and maximum pixel values are lower and upper limits. These values were calculated separately for each band and then used to stretch the contrast of the RGB image.

**Figure 4:**
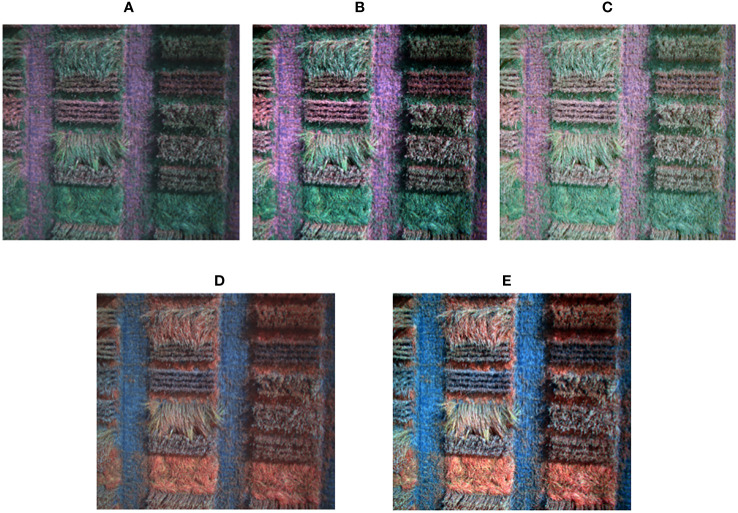
Compositions of enhanced RGB (3-2-1), (4-2-1). **(A)** Original RGB, **(B)** Haze & Gamma Adjustment, **(C)** Contrast stretching limits, **(D)** Customized image (4-2-1) before enhancement, **(E)** Customized image (4-2-1) enhanced composition.

New composite images were created by combining different bands than the traditional red, green, and blue ones. These customized images can potentially emphasize specific objects or features in the images that might not be visible in the visible spectrum. **Figure 4** compares a standard RGB image created using the traditional (3-2-1) combination and a customized image composed of bands (4-2-1).

#### 2.2.3 Data annotation

**Figure 5:**
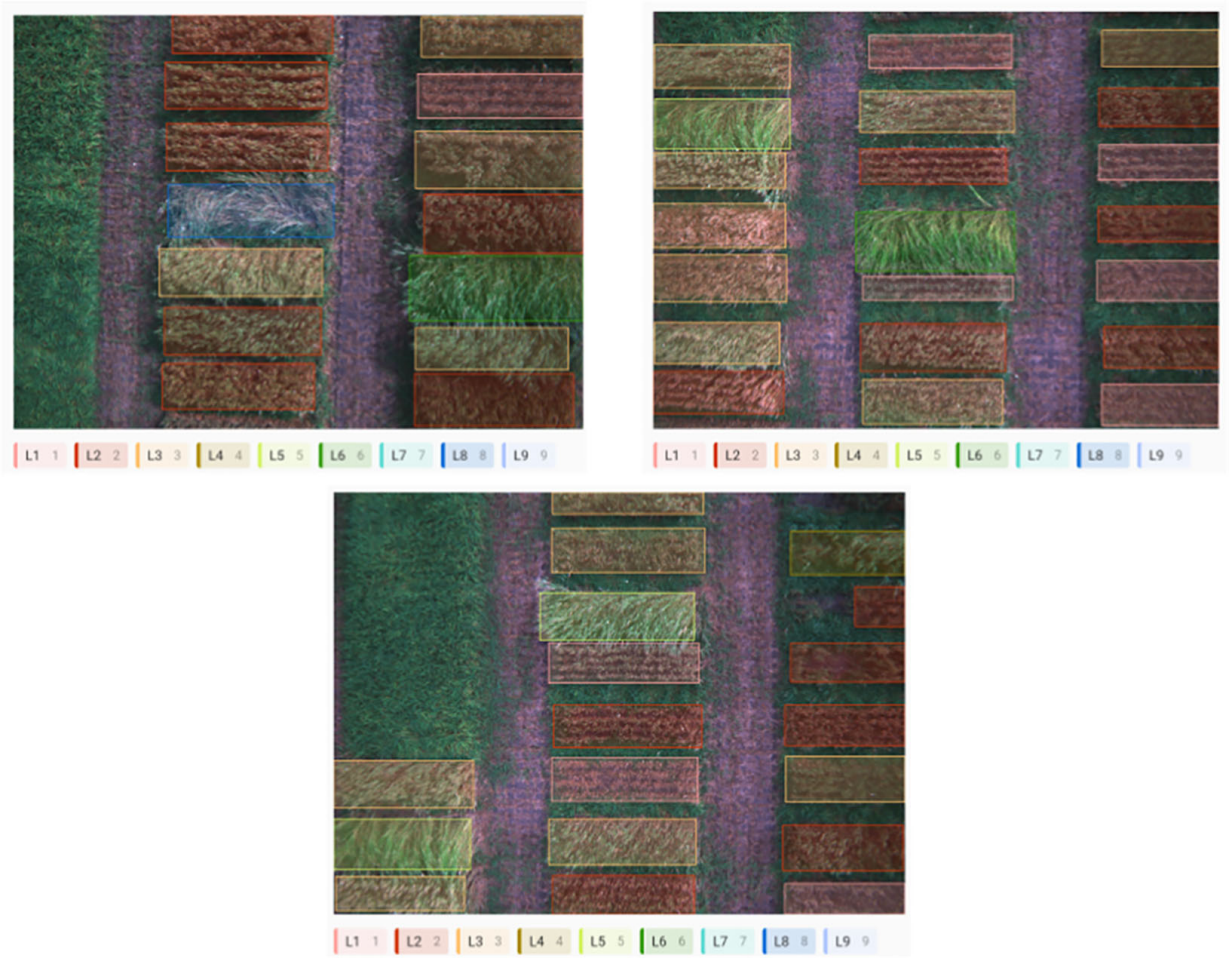
Process of labeling images for wheat lodging severity levels (L1-L9) using Label Studio.

The Label Studio tool (Tkachenko et al., 2020–2022) was used to label the images collected from the field plots based on the manual visual evaluation of lodging (1-9 scale, SASKSEED GUIDE, 2022), facilitating efficient data labelling. The nine categories of wheat lodging levels classified according to the severity of the damage were essential for precise monitoring of wheat growth and identifying areas needing corrective action. The model was trained to recognize these nine classes, with damage levels ranging from minimal to complete collapse. The labelling was done with high accuracy through the Label Studio tool, as demonstrated in **Figure 5**, ensuring the model is trained precisely. This enables the system to accurately differentiate between the various wheat lodging categories and assess the strong extent of plant straw strength.

### 2.3 Wheat lodging detection and classification

**Figure 6:**
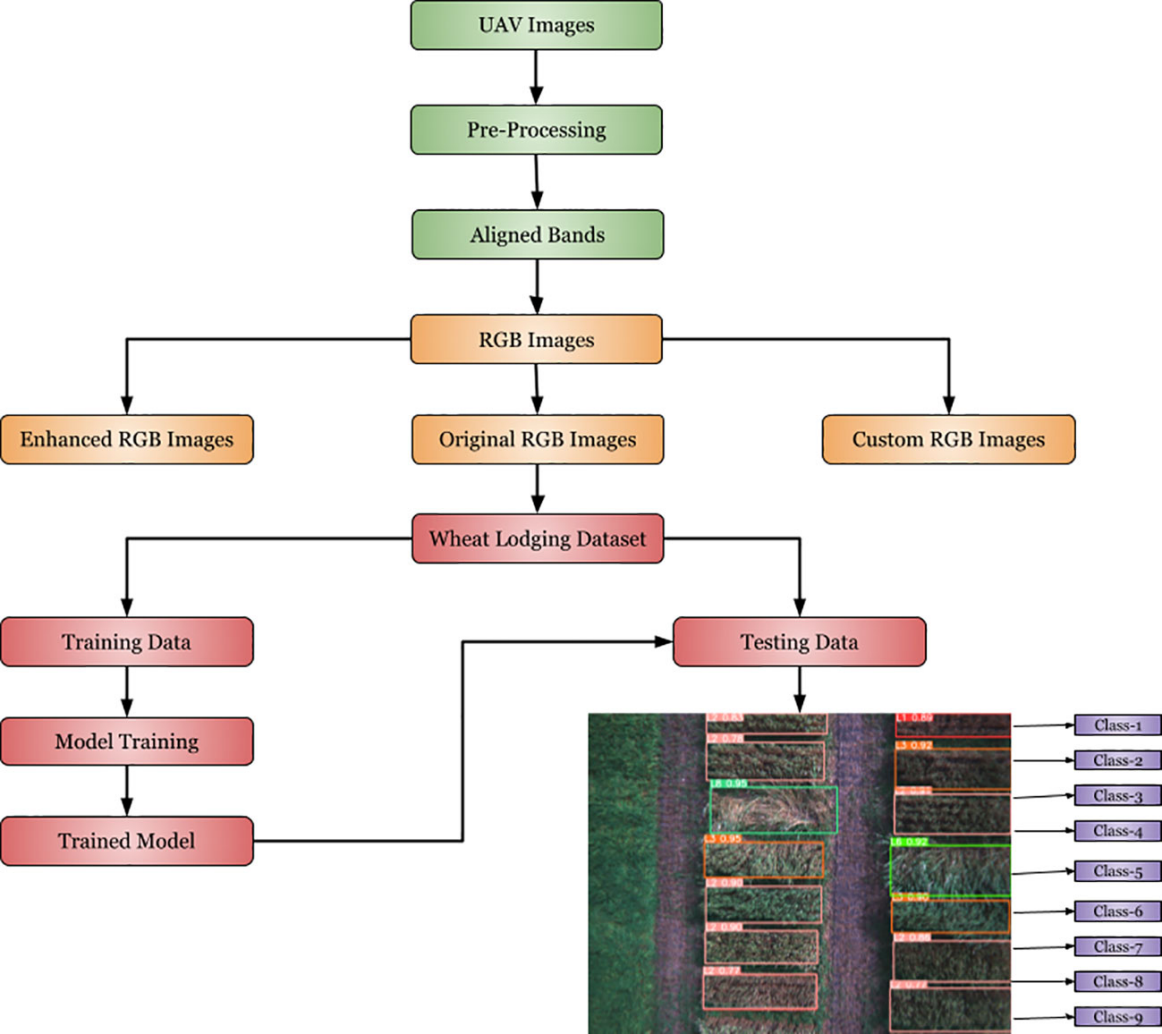
Framework flowchart of wheat lodging detection and classification.

The flowchart in **Figure 6** outlines the wheat lodging detection and classification process. Initially, a wheat lodging dataset was generated using the pre-processed multispectral images, as discussed in Section.2.1, and by labelling the ground-truth data. After creating the dataset, it was randomly shuffled and divided into training, validation, and testing sets. The training set was used for model training, and the model’s performance was assessed by utilizing the test set to produce the prediction and classification of wheat lodging results.

In this framework, we used a state-of-the-art YOLOv8 (Solawetz, 2023) model to produce the final prediction and classification output images. YOLOv8 requires minor modifications to meet the specific input and output image requirements. The key architectural enhancements and improvements include anchor-free detection, new convolutions and building blocks, neck modifications, mosaic augmentation adjustment, and improved accuracy. YOLOv8 introduces anchor-free detection, which predicts the center of an object directly, simplifying the Non-Maximum Suppression (NMS) process—a post-processing step to refine candidate detections after inference.

The model is highly effective for object detection and image classification. We applied YOLOv8 to detect and classify different types of lodging that can occur under field conditions. We trained the model on annotated images, specifying nine different classes (1-9 scale) of lodging, allowing YOLOv8 to identify and classify instances of wheat lodging effectively.

#### 2.3.1 Model architecture and detection process

**Figure 7:**
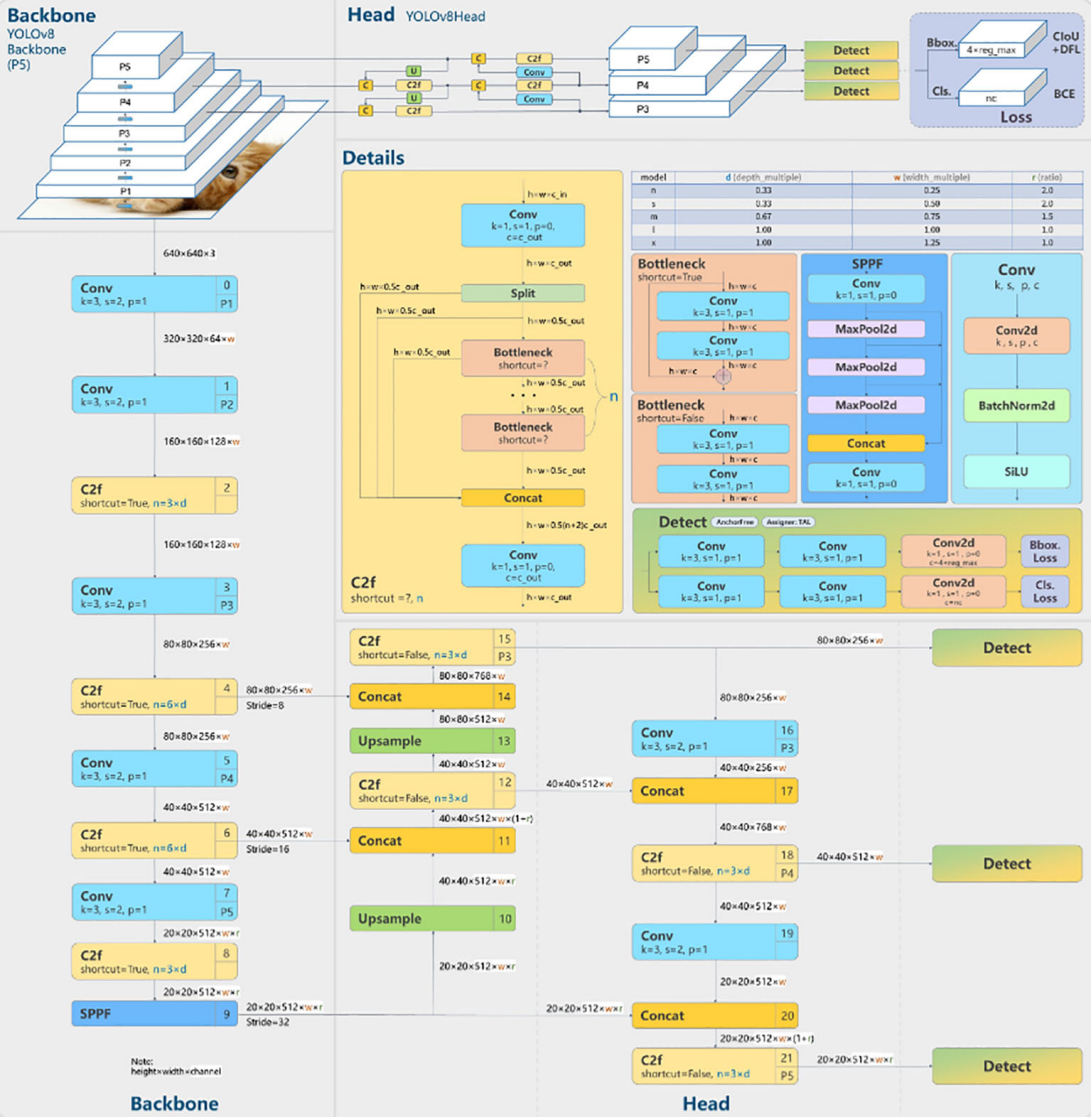
The architecture and visualisation of YOLOv8 (Contributors, 2023).

**Figure 7** (Contributors, 2023) provides a detailed overview of the architecture of YOLOv8. YOLOv8 is based on a similar framework as YOLOv5, with some modifications to the CSPLayer, now called the C2f module. The C2f module (cross-stage partial bottleneck with two convolutions) is responsible for merging high-level features with contextual information, ultimately enhancing the accuracy of object detection. YOLOv8 employs an anchor-free model with a decoupled head, allowing it to independently process tasks related to objectness, classification, and regression. This design ensures that each component can focus on its specific task, thereby improving the overall accuracy of the model Terven and Cordova-Esparza (2023).

In the output layer of YOLOv8, the sigmoid function is employed as the activation function for determining the objectness score, which represents the likelihood of a bounding box containing an object. For class probabilities, indicating the likelihood of an object belonging to a specific class, YOLOv8 utilizes the softmax function. Furthermore, YOLOv8 adopts Complete Intersection over Union (CIoU) and Distribution Focused Loss (DFL) loss functions for bounding box loss and binary cross-entropy for classification loss. These loss functions have proven effective in enhancing object detection performance, particularly when dealing with smaller objects.

We used the YOLOv8 object detection model to detect and classify wheat lodging severity in RGB input images, as shown in the following steps:

- **Input Image**: The process begins with an input image of a wheat field, where the goal is to detect and classify the severity levels or classes of wheat lodging within the classification scale.
- **Pre-processing:** The input image is resized and normalized to match the input size and format expected by the YOLOv8 model.
- **Model Inference**: The pre-processed image is then passed through the YOLOv8 model.
- **Raw Predictions**: The model generates raw predictions, including bounding box coordinates, class probabilities, and scores for potential wheat lodging classes.
- **Post-processing**: NMS is applied to the raw predictions to eliminate overlapping or duplicate detections.
- **Thresholding**: A confidence threshold is applied to the remaining predictions to eliminate detections with low confidence scores.
- **Output**: The resulting bounding boxes and class labels represent the detected wheat lodging classes within the input image. These overlay the original input image to visualize the wheat lodging detection results.

Applying the YOLOv8 object detection model in this framework allows for efficient and precise detection and classification of wheat lodging severity levels or classes in input images of wheat fields. This method offers valuable insights for crop management and yield estimation, contributing to the optimization of agricultural practices and informed decision-making for farmers and other stakeholders in the industry.

## 3 Results

### 3.1 Evaluation metrics

To quantitatively evaluate and analyze the detection and classification results, four evaluation metrics were used. The results are presented in graphs categorized as True Positive (TP), True Negative (TN), False Positive (FP), and False Negative (FN), based on whether the class is correctly or incorrectly identified. Precision, recall, F1 score, and mean Average Precision (mAP) are the chosen evaluation measures to assess the model’s performance.

#### 3.1.1 Precision

Precision measures the model’s reliability in identifying positive results among the actual positive ones. It is particularly useful when the cost of FP is high. Precision is also known as Positive Predictive Value (PPV). The precision is calculated by (7).


(7) $$Precision=\frac{TP}{TP+FP}$$

#### 3.1.2 Recall

Recall measures the number of actual positive instances correctly identified by the model (TP). It is used when FN has a high cost. The recall calculation is determined using (8).


(8) $$Recall=\frac{TP}{TP+FN}$$

#### 3.1.3 F1 Score

When there is a significant class imbalance (i.e., a large number of actual negatives), the F1 Score may be a better metric than precision or recall to achieve a balance between the two. The F1 Score was calculated by (9).


(9) $$F1=2\times \frac{Precision\times Recall}{Precision+Recall}$$

#### 3.1.4 Mean Average Precision

Mean Average Precision (mAP) is a widely used performance metric in object detection tasks that evaluates the precision of the model at various recall levels. It is the mean of the dataset’s AP values for each class. A higher mAP value generally indicates better performance of the model. The calculation is given by (10).


(10) $$mAP=\frac{1}{\left|classes\right|}\sum_{i=1}^{\left|classes\right|}AP_{i}$$

where |*classes*| is the number of classes in the dataset and *AP_i_* is the average precision for the *i*-th class.

#### 3.1.5 Confusion matrix

The confusion matrix is one of the metrics used to evaluate a model’s performance in an object detection task. To calculate the confusion matrix, it is important to understand the Intersection over Union (IoU) metric, also known as the Jaccard index. IoU measures the level of overlap between the Ground-Truth (GT) and the predicted bounding box, and it is calculated by dividing the area of overlap/intersection between the GT and predicted bounding box by the area of their union, as shown in (11).


(11) $$IoU=\frac{AreaofOverlap}{AreaofUnion}=\frac{TP}{TP+FP+FN}$$

### 3.2 Lodging field scenario

The wheat plots were classified into nine different lodging classes used in the study (**Table 1**), ranging from no lodging (Class 1) to severe lodging (Class 9). Each plot’s lodging severity was manually assessed using the rating scale used at AAFC-Swift Current’s wheat research program. The visual rating data for lodging was used in labelling during the model’s training and testing phases.

### 3.3 Model training

The study utilized the following software and hardware configuration: Windows 10 operating system, Python 3.9, PyTorch 2.0.0, and CUDA 11.7. The initial learning rate was set at 0.001, and the training set images had dimensions of 1427 × 1035. However, the image size was adjusted to 640 × 640 during model input. The training process involved 300 epochs with a batch size 16 and employed the ’YOLOv8s’ model, which boasts 11.2 million parameters. The dataset consisted of 2,000 multi-spectral images. After the data pre-processing step, where the multi-spectral images were transformed into RGB images, the dataset was randomly mixed and split into training (70%), validation (10%), and testing (20%) subsets.

### 3.4 Wheat lodging detection and classification results

**Figure 8:**
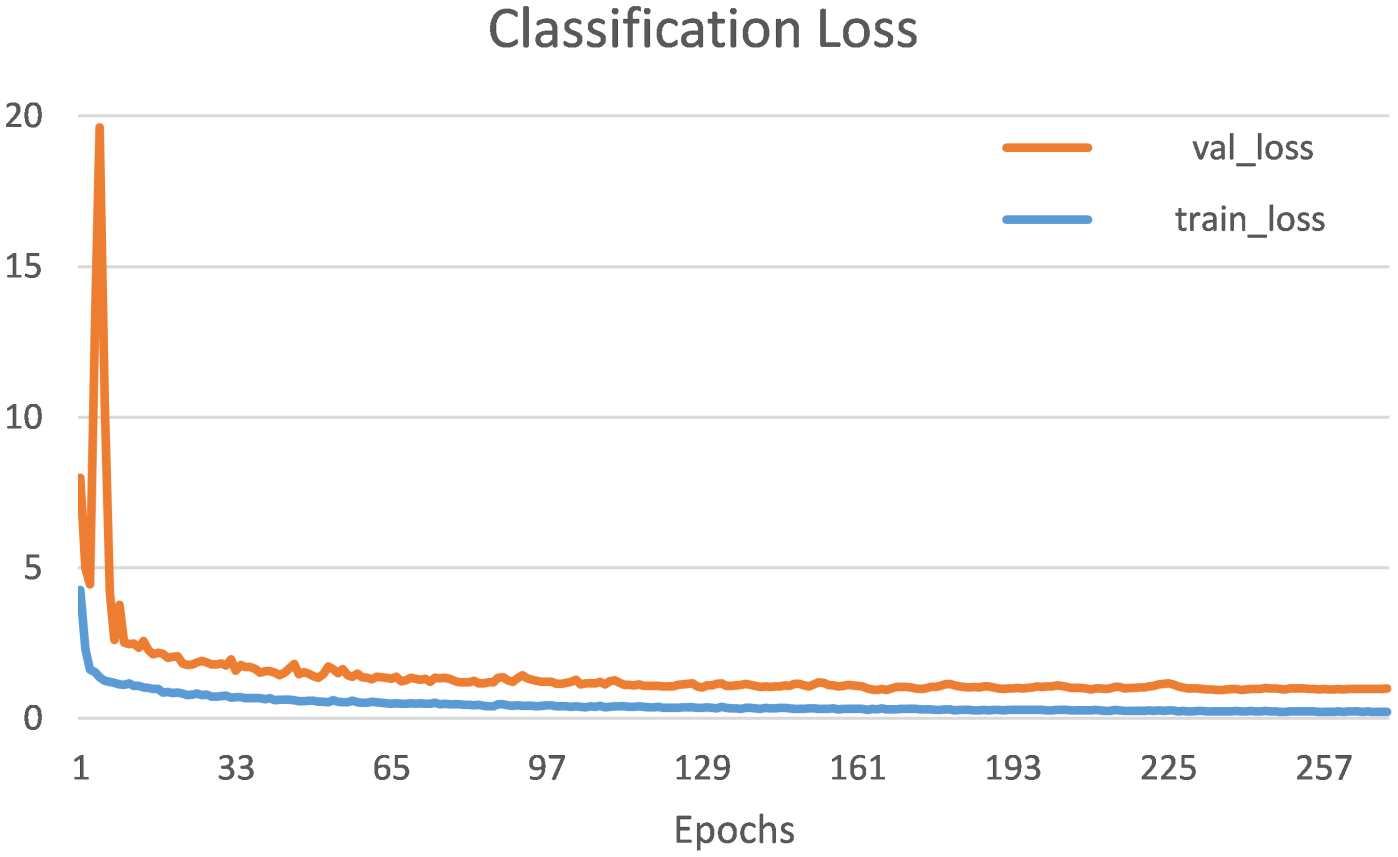
Training and validation classification loss.

**Figure 9:**
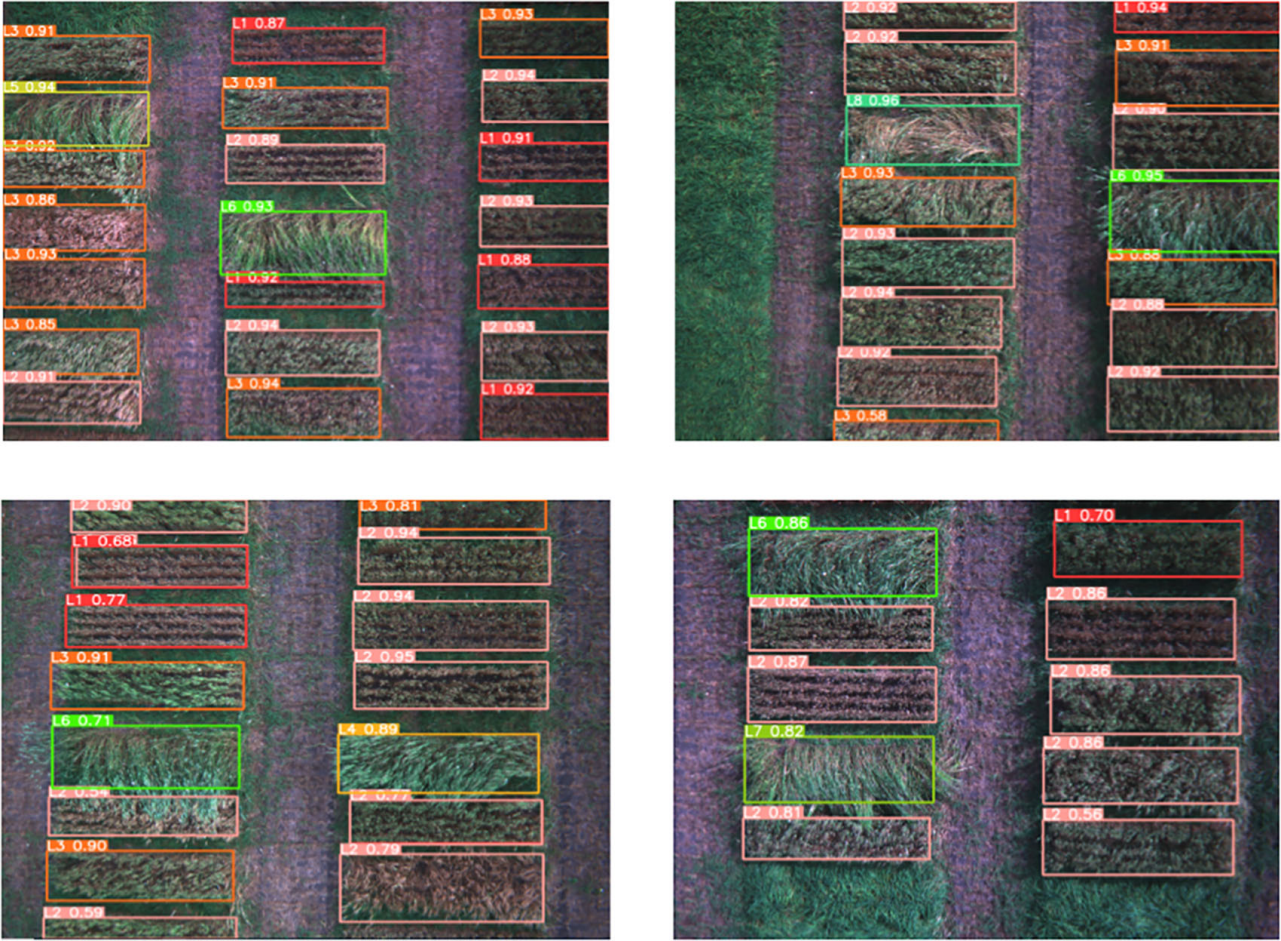
Framework detection and classification results.

During training the model over 300 epochs, we implemented an early stopping criterion to halt the training after 30 consecutive epochs with no observable improvement. The optimal weights, which are selected from the resulted epochs within this framework, were then used to construct the test model. **Figure 8** displays the classification loss for the training and validation sets. The graph demonstrates that the gap between the two sets decreased as the iterations increased and eventually reached a consistent state. The minimal difference in error between the training and validation sets indicates that the model’s variance is relatively low. In **Figure 9**, the wheat lodging detection and classification outcomes for the testing data are displayed. The model distinguished between nine distinct classes of wheat lodging. These outcomes demonstrate the framework’s outstanding forecasting and classification capabilities.

**Figure 10:**
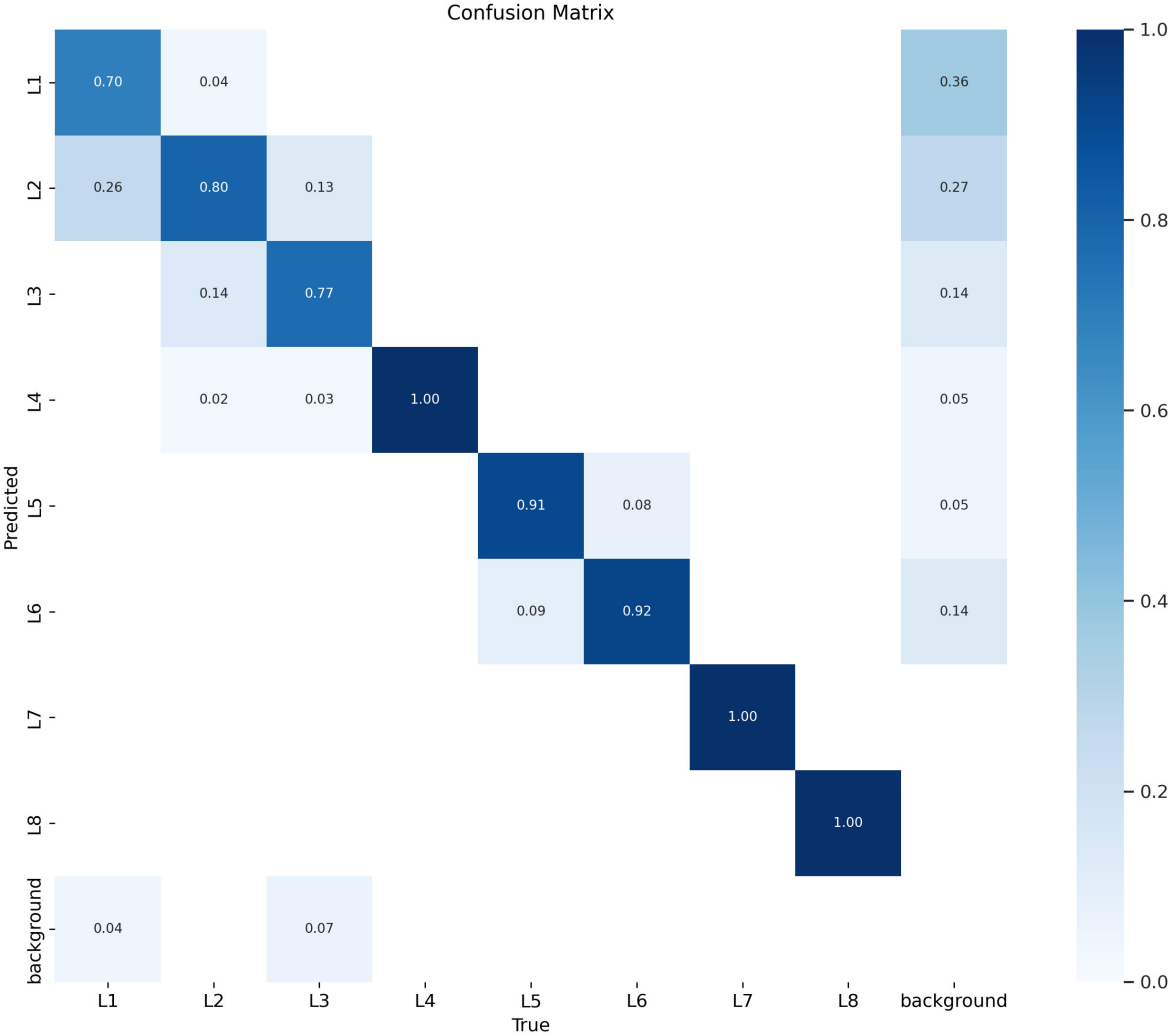
Confusion matrix of the model.

**Figure 11:**
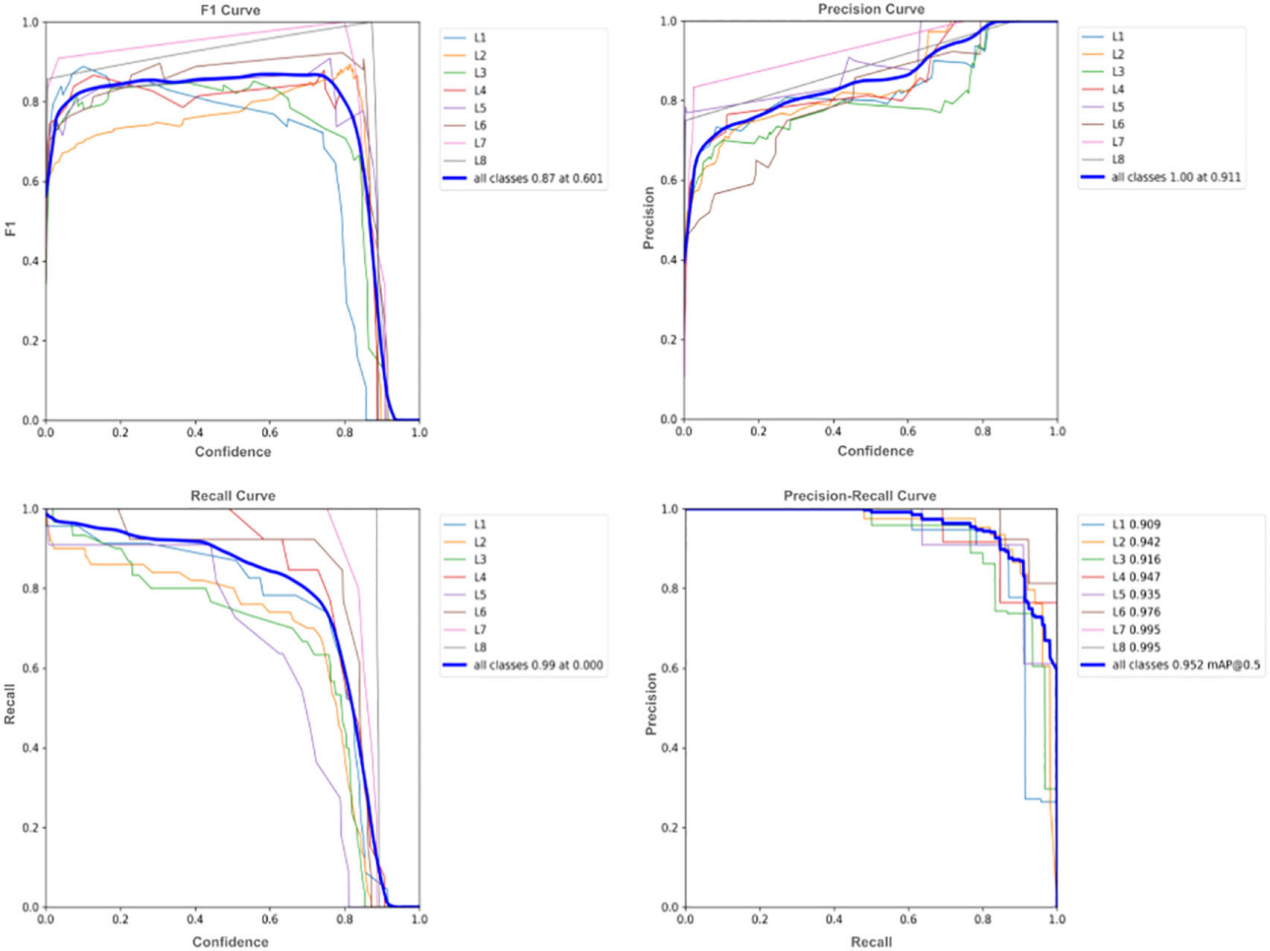
F1 curve, precision curve, recall curve, and precision-recall curve for model’s performance.

In this part of the research, we analyzed the evaluation metrics. The confusion matrix of the model is shown in **Figure 10** while **Figure 11** depicts evaluation metrics that offer a thorough evaluation of the results. By comparing the model’s predictions with the actual classes, the confusion matrix gives a useful indicator of the classification accuracy, which was used to classify different types of wheat lodging in this study. The IoU is used to evaluate the accuracy of the object detection task by quantifying the overlap between the predicted bounding boxes and the actual GT. The F1 curve displays an overall model performance of 0.87 at a 0.601 confidence level for all classes. This balance of precision and recall suggests the model’s effectiveness in predicting positive and negative cases. The precision curve presents a perfect score of 1.00 at a 0.911 confidence level for all classes, indicating the model’s exceptional ability to classify positive cases and reduce false positives. These high precision values ensure that the model’s predictions are correct. The recall curve reflects the model’s ability to identify true positive cases, with an outstanding value of 0.99 at a 0.000 confidence level for all classes. This means that the model correctly detected almost all positive cases, reducing the possibility of false negatives.

The Precision-Recall curve indicated a value of 0.952 at mAP@0.5 for all classes, demonstrating the balance between precision and recall at different confidence thresholds. This high value indicates a successful balance between precision and recall. Taken together, these curves provide a comprehensive assessment of the model’s performance in 4object detection, consistently demonstrating high levels of precision, recall, and F1 score, confirming the model’s effectiveness.

**Figure 12:**
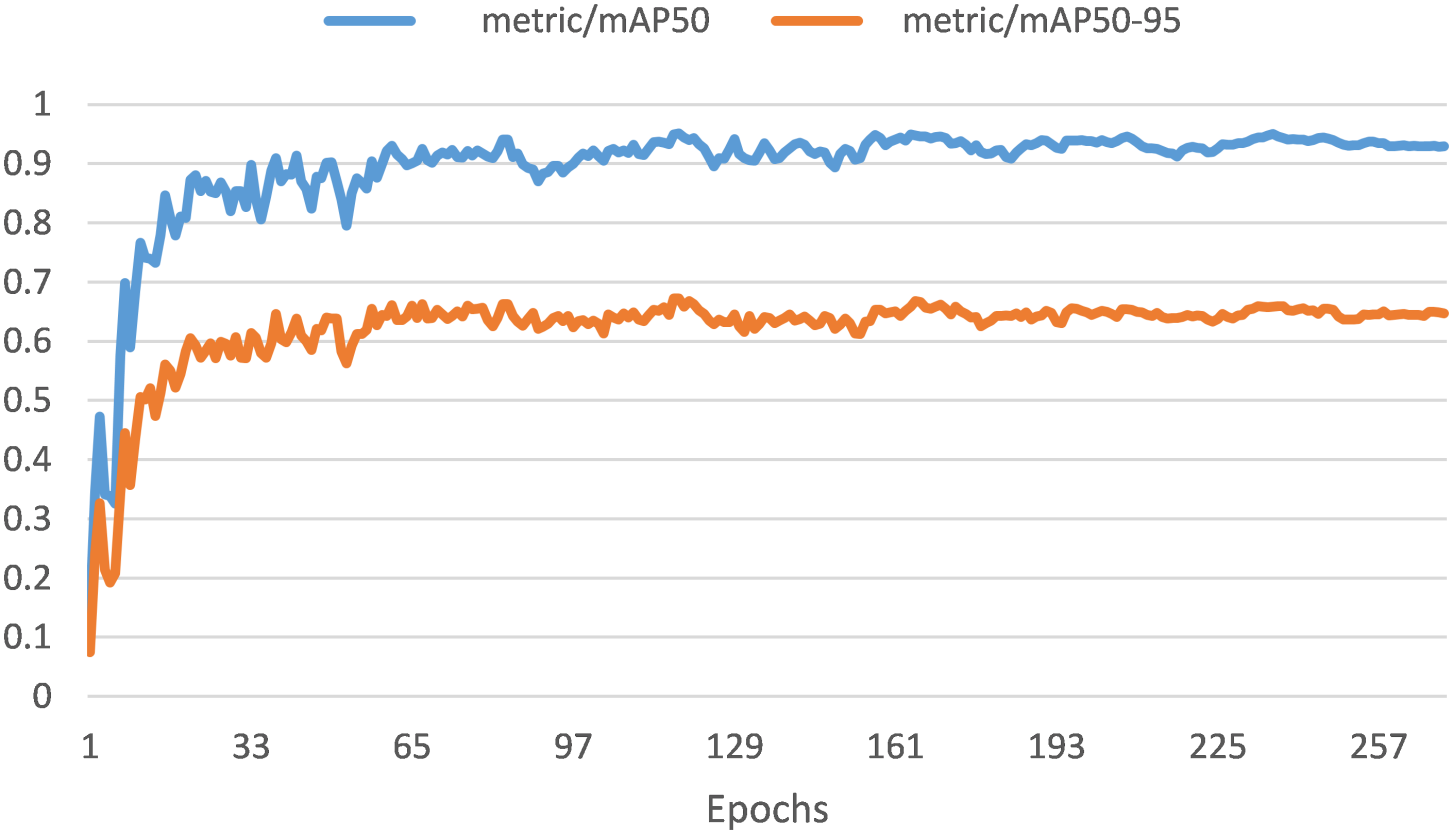
The mean average precision mAP50 (0.952%) and mAP50-95 (0.648%).

**Figure 12** presents the mAP for the current model, which offers a comprehensive evaluation of its overall precision performance in real-time. This metric was crucial for comparing different object detection models and identifying the most suitable one for a particular task. Higher mAP values indicated better model performance. Our study examined two mAP metrics: mAP @50and mAP@50-95. The model’s mAP@50is 0.952%, indicating a high precision value for a relatively generous IoU threshold. This suggests that the model performed well when a 50% overlap between predicted and actual bounding boxes is accepted. Meanwhile, the model’s mAP@50-95 is 0.641%, reflecting its precision across a range of IoU thresholds from 50% to 95%. This measure explains how well the model performs under stricter criteria when the precision of the bounding box predictions becomes increasingly important. Our reported mAP values provide valuable insights into the model’s overall performance, enabling informed decisions concerning its suitability for the specified framework.

### 3.5 Comparison

In comparing our LodgeNet framework with existing studies, it becomes evident that our approach offers several distinct advantages. While Jiang et al. (2022) focuses on using the SegFormer-B1 model for wheat lodging area calculation, achieving an accuracy of 96.56% using a DJI Phantom 4 Pro V2.0 UAV, equipped with RGB sensors. Tang et al. (2022) introduces the pyramid transposed convolution network (PTCNet) for large-scale wheat lodging extraction but relies primarily on GaoFen-2 satellite images. Finally, Zhang et al. (2022) explores UAV images at different mission heights for lodging detection, focusing on SVM and Resnet50 models.

Our LodgeNet leverages UAVs and deep learning, providing a more versatile, precise, and efficient method. Notably, we have multi-spectral UAV images and improved image quality through advanced techniques like ‘Haze & Gamma Adjustment’ and ‘Stretching Contrast Limits’ while utilizing the YOLOv8 deep learning algorithm for lodging detection. The results showcase a mean Average Precision (mAP) of 0.952% @0.5, indicating the remarkable accuracy of our LodgeNet. Additionally, we provide comprehensive range of lodging severity levels, each with competitive mAP scores, ranging from no lodging (Class 1) to severe lodging (Class 9), which is the missing component in all of the aforementioned articles. **Table 3** summarizes the comparison between LodgeNet and the referenced articles.

**Table 3:** LodgeNet comparison with referenced articles.

Study	Approach	Input Data	Performance	Lodging Severity
Jiang et al. (2022)	SegFormer-B1	RGB UAV images	Accuracy: 0.965%	No
Tang et al. (2022)	PTCNet	GaoFen-2 satellite images	F1, IoU: 0.853%, 0.743%	No
Zhang et al. (2022)	SVM+ResNet50	RGB UAV images	Accuracy: 0.676% (SVM), 0.672% (ResNet50)	No
**LodgeNet**	**YOLOv8**	**Multi-spectral UAV images**	**mAP: 0.952%**	**Comprehensive (Class 1-9)**

## 4 Discussion

Plant lodging is a major concern to wheat growers due to reductions in grain yield and deterioration in grain quality. Reducing the impact of lodging is required to improve the economic benefits of wheat farming. Both plant genetics and the growing environment can influence wheat lodging (Wu and Ma, 2016). Fast and accurate screening protocols for wheat lodging can help to identify the germplasm for use in improving lodging resistance in new cultivars. Accurate phenotyping using sensor-based technology and UAV (drones), and matched with machine with high precision learning can facilitate crop phenotyping for lodging resistance. The LodgeNet framework is an important step towards automated precision phenotyping of wheat lodging that explored deep learning algorithms combining visual rating, image processing, and data analysis to improve the accuracy with significant improvement.

Conventionally, lodging is recorded manually using a visual rating scale of 1-9 (SASKSEED GUIDE, 2022). The application of image-based detection of lodging with machine learning is a newer area that is still in the early stage of exploration. Other studies on crop lodging monitoring mainly employ traditional machine learning techniques such as k-Nearest Neighbours (kNN) (Peterson, 2009), Linear Discriminant Analysis (LDA) (Balakrishnama and Ganapathiraju, 1998), Random Forest (RF) (Cutler et al., 2007), Neural Network (NN) (Hagan and Demuth, 1999), and Support Vector Machine (SVM) (Noble, 2006), providing moderate accuracy in lodging detection. Zhang et al. (Zhang et al., 2022) employ SVM with color and texture features of UAV images for detecting wheat lodging. Liu et al. (Liu et al., 2018) use SVM to differentiate between lodging and non-lodging plants based on their color, texture, and thermal infrared features. In a study conducted in (Zhang et al., 2020), the GoogLeNet model is combined with RGB images obtained from UAV for wheat lodging detection. The approach resulted in an accuracy rate of over 90%, which necessitates improving the detection accuracy using other approaches (Marsland, 2015). Our method is significantly improved than many other published reports.

As shown in our study, deep learning network models are often better than many traditional machine learning algorithms due to their exceptional ability to learn complex patterns from large amounts of data, their proficiency in handling unstructured data such as images and text, and their capability to continuously learn and improve from experience. These deep learning approaches are increasingly used to improve the accuracy and efficiency of lodging detection cereals and other crops (Krizhevsky et al., 2017). Using computer vision and three deep learning methods (Faster R-CNN, YOLOv2, and RetinaNet), corn lodging information was extracted from UAV RGB images in (Hamidisepehr et al., 2020). In a similar approach, the extraction of wheat lodging from UAV RGB images was performed by (Li et al., 2019) using a comprehensive feature model based on two single features. The authors utilized a K-means algorithm to create a multi-temporal lodging area extraction method. (Chauhan et al., 2019b) used a multi-resolution segmentation algorithm and nearest neighbor classification algorithm with UAV multi-spectral images to distinguish different categories of lodging wheat, achieving an overall accuracy of 90%. Another study in (Cao et al., 2021) proposed a hybrid algorithm for extracting wheat lodging based on a watershed algorithm and adaptive threshold segmentation. Despite the high recognition accuracy of machine learning techniques in UAV lodging monitoring, their practical application is more complicated, requiring expert selection of features as pointed out by (Yu et al., 2023).

Existing approaches, such as ML and DL network models for wheat lodging detection, lack the inclusion of lodging severity information of wheat during detection and classification. The proposed framework addresses this limitation by focusing on wheat lodging severity levels, divided into nine classes ranging from no lodging (Class 1) to severe lodging (Class 9). To our knowledge, this is the first research work to detect and classify wheat lodging severity levels into nine different classes. The study finds that when classifying the severity of wheat lodging, the mAP50 for the model is recorded as 0.952% and 0.641% for mAP@50-95. These mAP values helped in real-time wheat monitoring of lodging severity levels, showing the effectiveness of the model and its capability in its application. The main contributions of the proposed framework as outlined below, can significantly impact various fields of investigation.

- The dataset used in the study comprises 2000 multi-spectral images.
- To ensure accurate detection and classification of wheat lodging resistance, an image registration technique was developed to align the bands of the multi-spectral images. This allows for generating a single RGB (3-2-1 combination) image, which was used for detecting and classifying wheat lodging resistance. This technique eliminates any potential distortion or misalignment in the images, resulting in a more precise analysis of the severity levels of wheat lodging.
- Image enhancement techniques were employed to improve the quality of the images, highlighting the important features for wheat lodging detection.
- A state-of-the-art deep learning technique called YOLOv8 was utilized to detect and classify wheat lodging severity levels. This method employs a CNN architecture for image object detection.
- An automated framework called ‘LodgeNet’ was developed to detect and classify the severity of wheat lodging class-wise. The model was trained on a large dataset of wheat lodging images, enabling it to accurately classify the severity level of the lodging detected in new images. ‘LodgeNet’ automatically displays the severity levels of wheat lodging class-wise, that could provide an efficient and automated solution for researchers and farmers to detect and classify the severity of wheat lodging accurately. ‘LodgeNet’ greatly improves the accuracy and efficiency of crop monitoring and management practices in agriculture.

**Figure 13:**
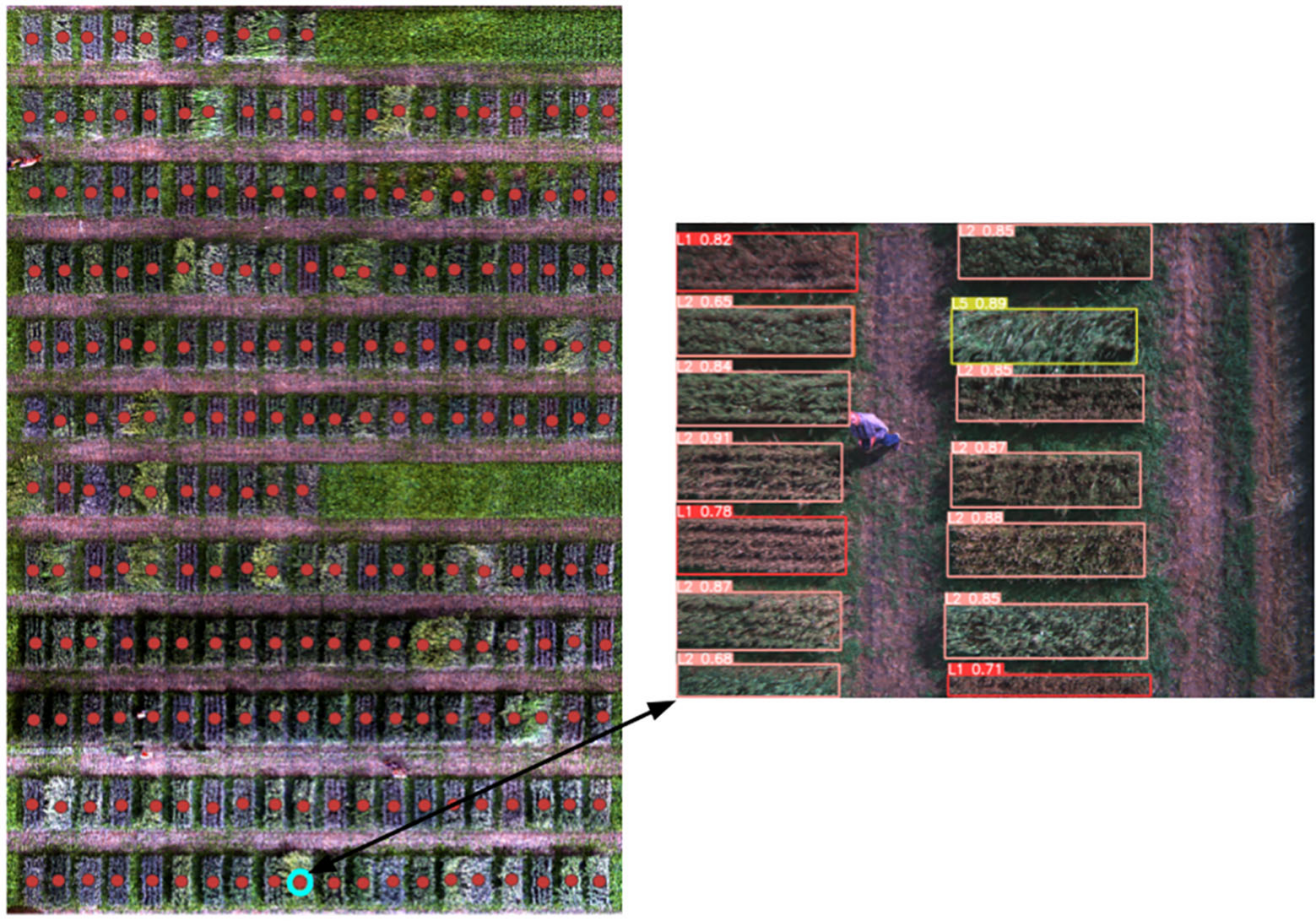
Clicking on a point, pop up the respective plot image with wheat lodging class.

**Figure 14:**
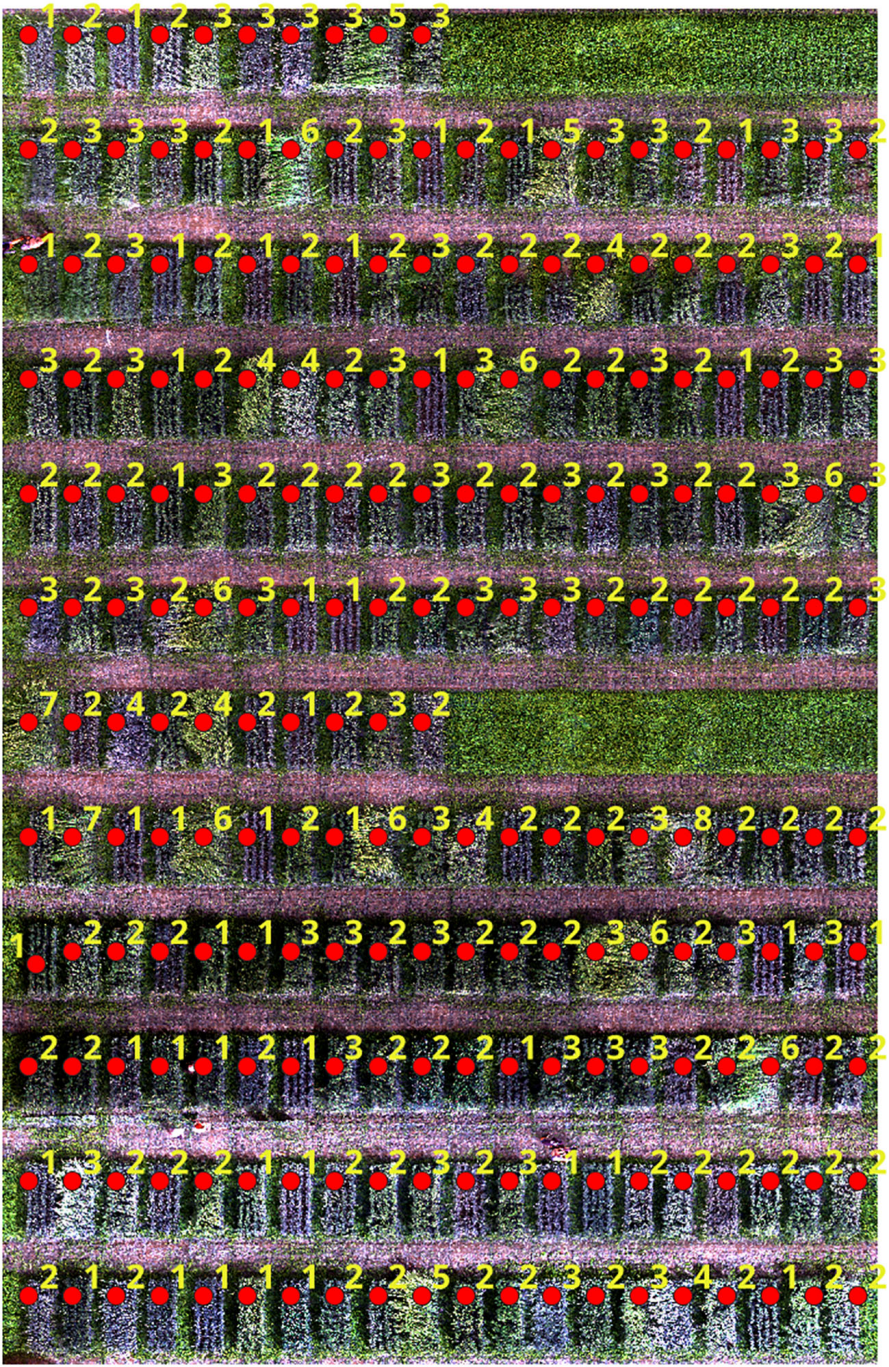
Lodging class alongside the marked points for each field plot.

The proposed framework also utilized QGIS to show the accurate detection and classification of wheat lodging. The framework was divided into two main steps. The first step of the framework involved inputting the orthomosaic of the wheat field, which then marks points on each plot of the field. Clicking on any point brings up the corresponding image of that plot with the detected and classified classes (severity levels) of wheat lodging. The second step presents the lodging class alongside the marked points for each field plot. An example of the detected and classified classes of wheat lodging can be seen in **Figures 13**, **14**. Compared to the manual rating, the accuracy of detection with the new approach seems reliable and could help breeders to take lodging notes with a single field image of several plots at different plant growth stages, saving time, resources and effort. This approach enables farmers to easily and efficiently monitor their crops, leading to improved management practices and increased crop yields.

## 5 Conclusion

In this research, we introduced LodgeNet, a framework that combines advanced techniques such as image registration, image enhancement, and deep learning for detecting wheat lodging with high precision that could classify the severity levels of wheat lodging, similar to the manual rating protocol used during wheat phenotyping. The development of this automated system could provide a streamlined and efficient solution for wheat breeders, farmers, crop surveyors, and other stakeholders, transforming how crops could be supervised and managed using modern high throughput approaches. The successful implementation of this framework offers immense potential to identify lodging resistance in wheat germplasm and develop better varieties for the farmers by facilitating more precise wheat monitoring and management practices that could boost grain yields and improve operational efficiency. Furthermore, the techniques and methodologies can be applied across various crops and in diverse agricultural scenarios. For example, our image registration and enhancement techniques have the potential to be utilized for identifying and categorizing diseases in wheat, simplifying wheat screening for different traits using single image data sets. Being simple but powerful, LodgeNet could also be tailored for other crops like rice, corn, or soybeans, providing researchers, crop estimators, and growers with an automated and efficient means of monitoring crop health and managing potential issues. This research could aid significant progress in crop management through image-based crop phenotyping, underscoring the importance of utilizing advanced technologies and methods in agriculture to enhance efficiency and productivity.

## Data availability statement

The raw data supporting the conclusions of this article will be made available by the authors, as per guidelines of the representative institutes. Further inquiries can be directed to the corresponding author.

## Author contributions

NA: Investigation, Methodology, Software, Validation, Writing – original draft. AM: Investigation, Methodology, Writing – original draft. AB: Methodology, Conceptualization, Funding acquisition, Project administration, Resources, Supervision, Writing – review & editing. JS: Conceptualization, Funding acquisition, Supervision, Writing – review & editing. YR: Conceptualization, Resources, Writing – review & editing. RC: Resources, Writing – review & editing.

## References

[B1] Abbas M. Saleem S. Subhan F. Bais A. (2020). Feature points-based image registration between satellite imagery and aerialimages of agricultural land. Turkish J. Electrical Eng. Comput. Sci. 28, 1458–1473. doi: 10.3906/elk-1907-92

[B2] Agisoft Metashape (2010). Discover intelligent photogrammetry with metashape. Available at: https://www.agisoft.com/.

[B3] Alawadi F. (2010). “Detection of surface algal blooms using the newly developed algorithm surface algal bloom index (SABI),” in Jr CRB Mertikas S. P. Neyt X. Velez-Reyes M., editors, Remote Sensing of the Ocean, Sea Ice, and Large Water Regions 2010 (International Society for Optics and Photonics (SPIE)), Vol. 7825. 45–58. doi: 10.1117/12.862096

[B4] Ali N. Ansari S. Halim Z. Ali R. H. Khan M. F. Khan M. (2019a). Breast cancer classification and proof of key artificial neural network terminologies. 2019 13th international conference on mathematics. Actuarial Science Comput. Sci. Stat (MACS) (IEEE), 1–6.

[B5] Ali N. Bonyadi M. R. Raja M. A. Akhtar J. Javed N. Ryan C. (2019b). “Fuscd-future smart car driver,” in 2019 22nd International Multitopic Conference (INMIC). 1–7 (IEEE).

[B6] Ali N. Halim Z. Hussain S. F. (2023). An artificial intelligence-based framework for data-driven categorization of computer scientists: a case study of world’s top 10 computing departments. Scientometrics 128, 1513–1545.

[B7] Al-Najjar H. A. Kalantar B. Pradhan B. Saeidi V. Halin A. A. Ueda N.. (2019). Land cover classification from fused dsm and uav images using convolutional neural networks. Remote Sens. 11, 1461.

[B8] Asad M. H. Bais A. (2020a). Weed detection in canola fields using maximum likelihood classification and deep convolutional neural network. Inf. Process. Agric. 7, 535–545. doi: 10.1016/j.inpa.2019.12.002

[B9] Asad M. H. Bais A. (2020b). Crop and weed leaf area index mapping using multi-source remote and proximal sensing. IEEE Access 8, 138179–138190. doi: 10.1109/ACCESS.2020.3012125

[B10] Balakrishnama S. Ganapathiraju A. (1998). Linear discriminant analysis-a brief tutorial. Institute Signal Inf. Process. 18, 1–8.

[B11] Berry P. Spink J. (2012). Predicting yield losses caused by lodging in wheat. Field Crops Res. 137, 19–26.

[B12] Cao W. Qiao Z. Gao Z. Lu S. Tian F. (2021). Use of unmanned aerial vehicle imagery and a hybrid algorithm combining a watershed algorithm and adaptive threshold segmentation to extract wheat lodging. Phys. Chem. Earth Parts A/B/C 123, 103016. doi: 10.1016/j.pce.2021.103016

[B13] Chauhan S. Darvishzadeh R. Boschetti M. Nelson A. (2020b). Discriminant analysis for lodging severity classification in wheat using radarsat-2 and sentinel-1 data. ISPRS J. photogrammetry Remote Sens. 164, 138–151. doi: 10.1016/j.isprsjprs.2020.04.012

[B14] Chauhan S. Darvishzadeh R. Boschetti M. Pepe M. Nelson A. (2019a). Remote sensing-based crop lodging assessment: Current status and perspectives. ISPRS J. photogrammetry Remote Sens. 151, 124–140. doi: 10.1016/j.isprsjprs.2019.03.005

[B15] Chauhan S. Darvishzadeh R. Lu Y. Boschetti M. Nelson A. (2020a). Understanding wheat lodging using multitemporal sentinel-1 and sentinel-2 data. Remote Sens. Environ. 243, 111804. doi: 10.1016/j.rse.2020.111804

[B16] Chauhan S. Darvishzadeh R. Lu Y. Stroppiana D. Boschetti M. Pepe M.. (2019b). Wheat lodging assessment using multispectral uav data. Int. Arch. Photogrammetry Remote Sens. Spatial Inf. Sci. 42, 235–240. doi: 10.5194/isprs-archives-XLII-2-W13-235-2019

[B17] Chauhan S. Darvishzadeh R. van Delden S. H. Boschetti M. Nelson A. (2021). Mapping of wheat lodging susceptibility with synthetic aperture radar data. Remote Sens. Environ. 259, 112427. doi: 10.1016/j.rse.2021.112427

[B18] Contributors M. (2023). MMYOLO: OpenMMLab YOLO series toolbox and benchmark. Available at: https://github.com/open-mmlab/mmyolo/tree/main/configs/yolov8.

[B19] Cutler D. R. Edwards T. C. Jr. Beard K. H. Cutler A. Hess K. T. Gibson J.. (2007). Random forests for classification in ecology. Ecology 88, 2783–2792. doi: 10.1890/07-0539.1 18051647

[B20] Dai X. Chen S. Jiang H. Nainarpandian C. (2023). “Rice lodging disaster monitoring method based on multisource remote sensing data,” in Wang Y., editor, International Conference on Geographic Information and Remote Sensing Technology (GIRST 2022) (International Society for Optics and Photonics (SPIE)) 12552, 1255224. doi: 10.1117/12.2667454

[B21] Das M. Bais A. (2021). Deepveg: Deep learning model for segmentation of weed, canola, and canola flea beetle damage. IEEE Access 9, 119367–119380. doi: 10.1109/ACCESS.2021.3108003

[B22] Fang J. Pan F. Lan Y. Lu L. Cao D. Yang D.. (2021). Wheat lodging area extraction using uav visible light remote sensing and feature fusion. Trans. Chin. Soc Agric. Eng. 37, 73–80. doi: 10.11975/j.issn.1002-6819.2021.03.009

[B23] Fischer R. Stapper M. (1987). Lodging effects on high-yielding crops of irrigated semidwarf wheat. Field Crops Res. 17, 245–258.

[B24] Hagan M. T. Demuth H. B. (1999). “Neural networks for control,” in Proceedings of the 1999 American control conference (cat. No. 99CH36251) (San Diego, CA, USA: IEEE), Vol. 3. 1642–1656. doi: 10.1109/ACC.1999.786109

[B25] Hamidisepehr A. Mirnezami S. V. Ward J. K. (2020). Comparison of object detection methods for corn damage assessment using deep learning. Trans. ASABE 63, 1969–1980. doi: 10.13031/trans.13791

[B26] He J. Zang Y. Luo X. Zhao R. He J. Jiao J. (2021). Visual detection of rice rows based on bayesian decision theory and robust regression least squares method. Int. J. Agric. Biol. Eng. 14, 199–206. doi: 10.25165/j.ijabe.20211401.5910

[B27] Jiang S. Hao J. Li H. Zuo C. Geng X. Sun X. (2022). Monitoring wheat lodging at various growth stages. Sensors 22, 6967. doi: 10.3390/s22186967 36146315 PMC9502829

[B28] Kang G. Wang J. Zeng F. Cai Y. Kang G. Yue X. (2023). Lightweight detection system with global attention network (gloan) for rice lodging. Plants 12, 1595. doi: 10.3390/plants12081595 37111819 PMC10145294

[B29] Kingsland K. (2020). Comparative analysis of digital photogrammetry software for cultural heritage. Digital App Archaeol Cultural Heritage 18, e00157. doi: 10.1016/j.daach.2020.e00157

[B30] Krizhevsky A. Sutskever I. Hinton G. E. (2017). Imagenet classification with deep convolutional neural networks. Commun. ACM 60, 84–90. doi: 10.1145/3065386

[B31] Li G. Zhang L. Song C. Peng M. Zhang Y. Han W. (2019). Extraction method of wheat lodging information based on multi-temporal uav remote sensing data. Trans. Chin. Soc. Agric. Machinery 50, 211–220. doi: 10.6041/j.issn.10001298.2019.04.02

[B32] Liu T. Li R. Zhong X. Jiang M. Jin X. Zhou P.. (2018). Estimates of rice lodging using indices derived from uav visible and thermal infrared images. Agric. For. meteorology 252, 144–154. doi: 10.1016/j.agrformet.2018.01.021

[B33] Marsland S. (2015). Machine Learning: An Algorithmic Perspective (1st ed.). (Chapman and Hall: CRC press). doi: 10.1201/9781420067194

[B34] Masuda R. Fujimoto S. Iida M. (2013). Suguri M. A method to detect the occurrence of rice plant lodging using wavelet transform. IFAC Proc. Volumes 46, 75–80. doi: 10.3182/20130828-2-SF-3019.00048

[B35] Modi R. U. Chandel A. K. Chandel N. S. Dubey K. Subeesh A. Singh A. K.. (2023). State-of-the-art computer vision techniques for automated sugarcane lodging classification. Field Crops Res. 291, 108797. doi: 10.1016/j.fcr.2022.108797

[B36] Moyroud N. Portet F. (2018). Introduction to QGIS (John Wiley Sons, Ltd), chap. 1. 1–17. doi: 10.1002/9781119457091.ch1

[B37] Noble W. S. (2006). What is a support vector machine? Nat. Biotechnol. 24, 1565–1567. doi: 10.1038/nbt1206-1565 17160063

[B38] Peterson L. E. (2009). K-nearest neighbor. Scholarpedia 4, 1883. doi: 10.4249/scholarpedia.1883

[B39] Pettorelli N. (2013). The normalized difference vegetation index (USA: Oxford University Press).

[B40] Pin˜era-Chavez F. Berry P. Foulkes M. Jesson M. Reynolds M. (2016). Avoiding lodging in irrigated spring wheat. i. stem and root structural requirements. Field Crops Res. 196, 325–336.

[B41] QGIS (2002). Available at: https://qgis.org/en/site/.

[B42] Raja R. Nguyen T. T. Slaughter D. C. Fennimore S. A. (2020). Real-time weed-crop classification and localisation technique for robotic weed control in lettuce. Biosyst. Eng. 192, 257–274. doi: 10.1016/j.biosystemseng.2020.02.002

[B43] SASKSEED GUIDE (2022). Published by Western Producer Publications. Available at: https://saskseed.ca/seed-guides/.

[B44] Setyawan R. A. Basuki A. Wey C. Y. (2020). “Machine vision-based urban farming growth monitoring system,” in 2020 10th Electrical Power, Electronics, Communications, Controls and Informatics Seminar (EECCIS). (Malang, Indonesia: IEEE) 183–187. doi: 10.1109/EECCIS49483.2020.9263449

[B45] Sewiko R. Sagala H. A. M. U. (2022). The use of drone and visible atmospherically resistant index (vari) algorithm implementation in mangrove ecosystem health’s monitoring. Asian J. Aquat. Sci. 5, 322–329.

[B46] Shah L. Yahya M. Shah S. M. A. Nadeem M. Ali A. Ali A.. (2019). Improving lodging resistance: using wheat and rice as classical examples. Int. J. Mol. Sci. 20, 4211. 31466256 10.3390/ijms20174211 PMC6747267

[B47] Shu M. Bai K. Meng L. Yang X. Li B. Ma Y. (2023). Assessing maize lodging severity using multitemporal uav-based digital images. Eur. J. Agron. 144, 126754. doi: 10.1016/j.eja.2023.126754

[B48] Solawetz J. (2023). What is yolov8? the ultimate guide. Jacob Solawetz, Francesco." Roboflow Blog, Jan 11, 2023. https://blog.roboflow.com/whats-new-in-yolov8/

[B49] Sun H. (2023). Crop Vegetation Indices. In Encyclopedia of Smart Agriculture Technologies (Cham: Springer International Publishing), 1–7.

[B50] Sun J. Zhou J. He Y. Jia H. Liang Z. (2023). Rl-deeplabv3+: A lightweight rice lodging semantic segmentation model for unmanned rice harvester. Comput. Electron. Agric. 209, 107823. doi: 10.1016/j.compag.2023.107823

[B51] Tang Z. Sun Y. Wan G. Zhang K. Shi H. Zhao Y.. (2022). Winter wheat lodging area extraction using deep learning with gaofen-2 satellite imagery. Remote Sens. 14, 4887.

[B52] Terven J. Cordova-Esparza D. (2023). A comprehensive review of yolo: From yolov1 to yolov8 and beyond. arXiv preprint arXiv:2304.00501.

[B53] Tkachenko M. Malyuk M. Holmanyuk A. Liubimov N. (2020–2022). Label Studio: Data labeling software. Open source software available from https://github.com/heartexlabs/label-studio.

[B54] Ullah H. S. Asad M. H. Bais A. (2021). End to end segmentation of canola field images using dilated u-net. IEEE Access 9, 59741–59753. doi: 10.1109/ACCESS.2021.3073715

[B55] Vargas J. Q. Khot L. R. Peters R. T. Chandel A. K. Molaei B. (2019). Low orbiting satellite and small uas-based highresolution imagery data to quantify crop lodging: a case study in irrigated spearmint. IEEE Geosci. Remote Sens. Lett. 17, 755–759. doi: 10.1109/LGRS.2019.2935830

[B56] Wang A. Zhang W. Wei X. (2019). A review on weed detection using ground-based machine vision and image processing techniques. Comput. Electron. Agric. 158, 226–240. doi: 10.1016/j.compag.2019.02.005

[B57] Wen J. Yin Y. Zhang Y. Pan Z. Fan Y. (2023). Detection of wheat lodging by binocular cameras during harvesting operation. Agriculture 13, 1–15. doi: 10.3390/agriculture13010120

[B58] Wu W. Ma B. L. (2016). A new method for assessing plant lodging and the impact of management options on lodging in canola crop production. Sci. Rep. 6, 1–17. doi: 10.1038/srep31890 27552909 PMC4995411

[B59] Xie T. Li J. Yang C. Jiang Z. Chen Y. Guo L.. (2021). Crop height estimation based on uav images: Methods, errors, and strategies. Comput. Electron. Agric. 185, 106155. doi: 10.1016/j.compag.2021.106155

[B60] Yang B. Zhu Y. Zhou S. (2021). Accurate wheat lodging extraction from multi-channel uav images using a lightweight network model. Sensors 21, 6826. doi: 10.3390/s21206826 34696038 PMC8538952

[B61] Yu J. Cheng T. Cai N. Lin F. Zhou X. G. Du S.. (2022). Wheat lodging extraction using improved unet network. Front. Plant Sci. 13. doi: 10.3389/fpls.2022.1009835 PMC9563998 36247550

[B62] Yu J. Cheng T. Cai N. Zhou X. G. Diao Z. Wang T.. (2023). Wheat lodging segmentation based on Lstm_PSPNet deep learning network. Drones 7 (2), 143. doi: 10.3390/drones7020143

[B63] Zaji A. Liu Z. Xiao G. Bhowmik P. Sangha J. S. Ruan Y. (2023). Wheat spikes height estimation using stereo cameras. IEEE Trans. AgriFood Electron. 1 (1), 15–28. doi: 10.1109/TAFE.2023.3262748

[B64] Zhang Z. Flores P. Igathinathane C. L Naik D. Kiran R. Ransom J. K. (2020). Wheat lodging detection from uas imagery using machine learning algorithms. Remote Sens. 12, 1838. doi: 10.3390/rs12111838

[B65] Zhang Z. Igathinathane C. Flores P. Mathew J. Ransom J. Ampatzidis Y.. (2022). UAV Mission Height Effects on Wheat Lodging Ratio Detection (Singapore: Springer Nature Singapore), 73–85. doi: 10.1007/978-981-19-2027-15

